# Coming to a good end after a difficult Covid-19 driven year

**DOI:** 10.1007/s00187-020-00313-0

**Published:** 2021-01-27

**Authors:** Thomas W. Guenther

**Affiliations:** Technische Universität Dresden, Dresden, Germany

The ending year 2020 was heavily driven by the Covid-19 pandemic. Also institutions of higher education were challenged to adjust teaching and research to the consequences of the pandemic. Teaching had to be converted from in-class offers to online productions challenging the didactical and technical capabilities of professors and lecturers. Research had to consider the hygenic limitations in contacting respondents and participants for example in field studies or on-site experiments. I know some colleagues who actually stopped collecting data.

But also our private situations changed a lot with many of us working in home office in sometimes too small flats and houses. In lockdowns, as in many of the Western countries, colleagues additionally had to homeschool their kids and to take care of the very young ones. However, time for commuting also reduced to the three minutes from the sleeping room to the home office. The feeling of being locked in caused emotional stress for many of us.

However, there is also light at the end of the tunnel. On the one hand, there is hope that the Covid-19 pandamic might flatten out by the end of the summer when vaccines are available for all of us. On the other hand, the Covid-19 crisis, as most of the crises, also pushed innovative disruptions which normally would have taken much longer. Digitalisation of administrative, teaching and research processes strongly unfolded. New formats of teaching, but also in research were developed. I had the chance to participate in U.S. brown bag sessions without leaving my home office, something which had not been possible before. Conferences and workshops were digitalized leaving me asking whether I would travel around the world as much as before the crisis. However, there are also tremendous shortcomings. I miss the chats with colleagues at conference dinners or at workshop coffee breaks. I also miss the short smalltalk with students in class. And, not only sometimes, I also miss the direct and personal communication with my colleagues.

Despite all these challenges and fluctuations, for the Journal of Management Control the year 2020 ends relatively normal with an issue consisting of four very interesting research papers.

*Patrick Velte* and *Martin Stawinoga* explore in a systematic literature review whether chief sustainability officers and CSR committees influence CSR-related outcomes. Based on 48 quantitative peer-reviewed empirical studies and informed by legitimacy theory, stakeholder theory and upper-echelons theory they investigate the influence of both CSR committees and CSOs on three CSR measures mainly analysed in empirical-quantitative research: CSR reporting, CSR assurance, and CSR performance.

Festivals are an important part of the popular culture and have increased in popularity in recent decades. However, they remain relatively unexplored in the accounting literature. *Per Ståle Knardal* and *Trond Bjørnenak* investigate informed by upper echelons theory and based on a survey of 61 festival managers from 40 festivals the use of budgets in festivals. They explore how individual and observable characteristics of festival managers are associated with variations in the use of budgets. The authors find that festival budgets are particularly important in the planning and coordination process but used less frequently for ex post evaluations. A positive association between a business educational background and the use of budgets for most purposes, with the exceptions of performance evaluation and reward, is supported.

Do management controls form packages or systems? A question which has been heavily discussed in management control research. *Hakim Lyngstadaas* explores this question for the field of working capital management in listed U.S. manufacturing firms. His study examines how working capital management packages can lead to higher financial performance by exploring the formation, importance, and systematic interdependencies within and between working capital management packages. The data set consists of 589 U.S. listed manufacturing firms over the fiscal period 2012–2019. He combines fuzzy set Qualitative Comparative Analysis (fsQCA) and panel data regression. Overall, eleven effective working capital management packages are found to be associated with high financial performance. Six of them constitute unique and empirically important packages and are also identified as systems.

Finally, *Kevin Baird, Amy Tung* and *Sophia Su* provide an empirical insight into the mediating role of the quality of performance appraisal systems using four quality dimensions (trust, clarity, communication and fairness) on the association between the level of employee empowerment and organisational performance. Data were collected from 203 Australian lower level managers using an online survey. The authors find that while employee empowerment is positively associated with all four dimensions of the quality of the performance appraisal system, only one specific dimension, trust, mediates the association between employee empowerment and organisational performance for both financial and non-financial performance.

Dresden, December 2020

For the team of editors

Thomas W. Guenther

Frank Verbeeten

Managing editors

